# Supplementary material for: Prognostic Value of Tumor Size in Resected Stage IIIA-N2 Non-Small-Cell Lung Cancer
Source: J Clin Med. 2020 May 1;9(5):1307. doi: 10.3390/jcm9051307 (PMC7290400; doi:10.3390/jcm9051307)
Supplement: Supplementary file 1 [file jcm-09-01307-s001.pdf]

Supplementary Materials

Prognostic Value of Tumor Size in Resected Stage IIIA-N2 Non-Small-Cell Lung Cancer

Chih-Yu Chen [1,2], Bing-Ru Wu [1,2], Chia-Hung Chen [1,2], Wen-Chien Cheng [1,2], Wei-Chun Chen [1,3,4], Wei-Chih Liao [1,2,4,*], Chih-Yi Chen [5], Te-Chun Hsia [1,3,4], Chih-Yen Tu [1,2]

[1] Division of Pulmonary and Critical Care Medicine, Department of Internal Medicine, China Medical University Hospital, Taichung, Taiwan
[2] School of Medicine, China Medical University, Taichung, Taiwan
[3] Department of Respiratory Therapy, China Medical University, Taichung, Taiwan
[4] Hyperbaric oxygen therapy center, China Medical University Hospital, Taichung, Taiwan
[5] Department of Surgery, Chung Shan Medical University Hospital, Taichung, Taiwan
* Correspondence: weichih.liao@gmail.com; Tel.: +886-4-2205-2121 EXT.4661 (W.-C.L.)

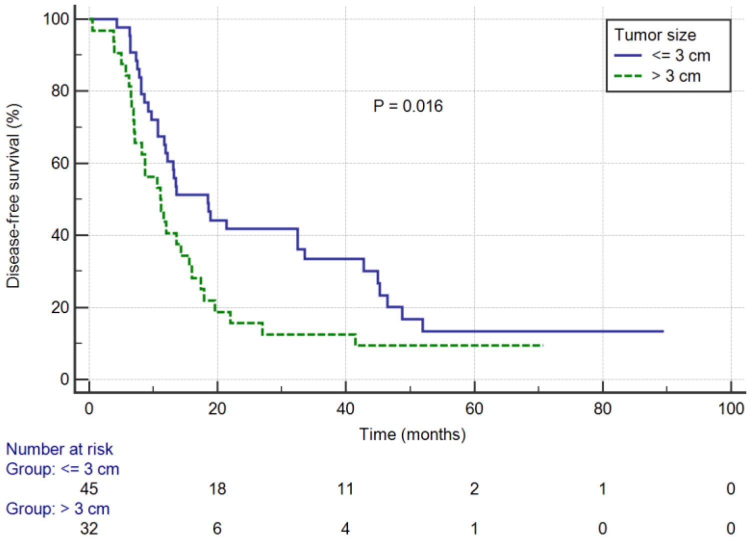

Figure S1. Disease-free survival curves of patients stratified by tumor size.

Table S1. Univariate analysis of factors associated with disease-free survival.

**Parameter**	**Hazard ratio (95% CI)**	**P value**
Age (≥65 y versus <65)	1.144 (0.680-1.923)	0.612
Gender (male versus female)	0.900 (0.543-1.490)	0.681
Ever smoker (yes versus no)	0.884 (0.523-1.494)	0.645
Family history of cancer (yes versus no)	0.664 (0.345-1.278)	0.220
Performance status (ECOG 1 versus ECOG 0)	1.278 (0.776-2.104)	0.335
Clinical N2 (yes versus unsuspected)	1.560 (0.950-2.563)	0.079
Limited resection (yes versus anatomical)	1.797 (0.853-3.788)	0.123
VATS (yes versus open thoracotomy)	0.763 (0.463-1.258)	0.289
Tumor size (≤3 versus 3-5)	0.543 (0.328-0.900)	0.018
Histology (adenocarcinoma versus others)	1.158 (0.617-2.175)	0.648
Differentiation (poor versus others)	1.090 (0.651-1.825)	0.742
CEA (≥3 versus <3)	1.796 (0.985-3.276)	0.056
Visceral pleural involvement (yes versus no)	1.366 (0.831-2.248)	0.219
Lymphovascular permeation (yes versus no)	1.182 (0.582-2.400)	0.643
Perineural invasion (yes versus no)	0.846 (0.417-1.718)	0.643
N2 ratio (≥40% versus <40%)	1.370 (0.814-2.304)	0.236
Central location (yes versus peripheral)	1.242 (0.750-2.055)	0.399
Lower lobe location (yes versus upper or middle)	0.988 (0.593-1.644)	0.961
Multiple N2 station (yes versus no)	1.220 (0.703-2.120)	0.480
Induction therapy (yes versus no)	0.568 (0.258-1.250)	0.160
Adjuvant therapy (yes versus no)	1.264 (0.617-2.591)	0.523
Postoperative radiotherapy (yes versus no)	0.648 (0.366-1.145)	0.135

Variables with P values of less than 0.2 were tested in multivariate analysis. ECOG, Eastern Cooperative Oncology Group; VATS, video-assisted thoracoscopic surgery; CEA, carcinoembryonic antigen.

Table S2. Multivariate analysis of factors associated with disease-free survival.

**Parameter**	**Hazard ratio (95% CI)**	**P value**
Clinical N2 (yes versus unsuspected)	2.525 (1.340-4.757)	0.004
Limited resection (yes versus anatomical)	1.442 (0.547-3.803)	0.459
Tumor size (≤3 versus 3-5)	0.451 (0.235-0.865)	0.017
CEA (≥3 versus <3)	1.810 (0.914-3.588)	0.089
Induction therapy (yes versus no)	0.577 (0.185-1.800)	0.343
Postoperative radiotherapy (yes versus no)	0.889 (0.420-1.882)	0.758

CEA, carcinoembryonic antigen.
